# Supplementary material for: Genetic variability and population structure of Ethiopian chickpea (Cicer arietinum L.) germplasm
Source: PLoS One. 2021 Nov 29;16(11):e0260651. doi: 10.1371/journal.pone.0260651 (PMC8629288; doi:10.1371/journal.pone.0260651)
Supplement: Attachment — Submitted filename: Response to Reviewers.doc [file pone.0260651.s002.doc]

**Date**: Aug 26 2021

**To**: "PLOS ONE" [plosone@plos.org](mailto:plosone@plos.org)

**From:** "Sintayehu Admas" [sintayahu.2010@gmail.com](mailto:sintayahu.2010@gmail.com)

**Subject:** Response to Reviewers

This letter is written to give responses for each points raised by the reviewers and journal editors point by points in tabular forms.

| **No** | **Reviewers comments** | **Author Responses** |
| --- | --- | --- |
| **Journal Requirements** (Blue font color is used for track) | | |
| 1 | PLOS ONE's style requirements | Addressed |
| 2 | Remove funding information from Acknowledgements Section | Addressed |
| 3 | Data availability | All the data are included in the manuscript |
| 4 | Reference update | Three references are cited in the text and added in the references list (i.e, [3], [4] and [30]) to accommodate the comments given by reviewer 1. |
| **Reviewer 1** (Green font color is used for track) | | |
| 1 | Reasons for no great differences between accessions based on geographical origin | It is raised an interesting issue. Many research report of cultivated crops shows that within population is more variable that among populations of different geographic origin in Ethiopia and similar result also was obtained in chickpea landraces. This is because of the informal seed exchange system of the country. Farmers have been practicing farmers-to-farmers seed exchange mechanism for a long period of time. Or, farmers sometimes buy seeds from the markets which might come from other places by merchants. In addition, farmers move from one place to other places for farming and as the same time they carry seeds with them to new areas. These activities facilitates gene flow among population (see table Table 6) |
| 2 | Better to Concentrate on landraces | To clarify more, 152 chickpea genotypes were used for the study i.e. 133 were lines selected from Ethiopian landraces, 8 were released varieties (improved varieties) and 6 were ICARDA breeding lines. So, we gave more emphasis to the landraces for this study. However, we prefere to use Ethiopian genotypes than landraces for this specific work because the landraces were splited into homogenous line which may create confusion to you. |
| 3 | Check if there is gene flow and introgression happened also within local varieties | Yes, there s gene flow observed within the landraces as since the value of Nm is above 1 (See Table 6) |
| 4 | Map showing the distribution of landraces | Addressed (Fig 1) |
| 5 | Clustering genotypes based on altitude issue | Regarding altitude based clustering. The study was targeted at the major chickpea growing areas of Ethiopia. The altitudinal variations among the collection sites were narrow, which makes difficulty to group accession based on altitude see altitudinal effects to the genotypes. In addition, the informal seed exchange systems have been practiced for centuries among traditional farmers in Ethiopia, we got similar results that of geographical origin and we did not present the result to limit the size of the paper. |
| 6 | Use of picture with poor resolution power | All Figs are addressed except Fig 1 (now Fig 2) because silver stained gel picture have most of the time have less resolution power. I can remove if it is not visible. |
| 7 | Give more reasoning for population structure | Addressed |
| 8 | The characteristics of desi and kabuli type chickpeas | Addressed in the introduction part |
| **Reviewer 2** (Red font color is used for track) | | |
| 1 | Vague sentences  Line 65 to 69; 79 to 83; 448 to 451(in older version) | Sentence that might have created misunderstanding or vague have been reviewed |
| 2 | Identify the landraces | Addressed |
| 3 | The dendrogram did not indicate any clear divisions between desi and kabuli type chickpea | Addressed |
| 4 | The material distinction in desi and kabuli has not been addressed in materials and methods | Addressed |
| 5 | Insert a figure of the geographical distribution of the material used. | Addressed (Fig 1) |
| 6 | I suggest a better quality of results of the images used, in particular Fig1 and Fig3 | All Figs are addressed except Fig 1 (now Fig 2) because silver stained gel picture have most of the time have less resolution power. I can remove if it is not visible. |

Kind regards,

Sintayehu Admas, PhD candidate
Corresponding Authors
